# Bimodal Gene Expression and Biomarker Discovery

**DOI:** 10.4137/cin.s3456

**Published:** 2010-02-04

**Authors:** Adam Ertel

**Affiliations:** Kimmel Cancer Center, Department of Cancer Biology, Thomas Jefferson University, Philadelphia, PA, USA. Email: adam.ertel@jefferson.edu

**Keywords:** biomarkers, cancer, genomics, bimodal, gene expression microarrays

## Abstract

With insights gained through molecular profiling, cancer is recognized as a heterogeneous disease with distinct subtypes and outcomes that can be predicted by a limited number of biomarkers. Statistical methods such as supervised classification and machine learning identify distinguishing features associated with disease subtype but are not necessarily clear or interpretable on a biological level. Genes with bimodal transcript expression, however, may serve as excellent candidates for disease biomarkers with each mode of expression readily interpretable as a biological state. The recent article by Wang et al, entitled “The Bimodality Index: A Criterion for Discovering and Ranking Bimodal Signatures from Cancer Gene Expression Profiling Data,” provides a bimodality index for identifying and scoring transcript expression profiles as biomarker candidates with the benefit of having a direct relation to power and sample size. This represents an important step in candidate biomarker discovery that may help streamline the pipeline through validation and clinical application.

High-throughput gene expression assays are capable of generating large-scale datasets that are useful in gaining insight to healthy biological systems, disease phenotypes, and biomarkers that are representative of these phenotypes. The recent publication by Wang et al^1^ provides a sound approach for mining through these expression datasets to identify and rank a class of genes, with bimodal expression profiles, that may serve as ideal biomarker candidates. Biomarkers that correspond with disease phenotypes are a useful tool for the diagnosis, treatment, and prognosis of disease. Cancer, as a heterogeneous disease, has many subtypes that respond differently to treatment and have different overall prognosis.^2^ Biomarkers with accurate and reliable assays can be useful in identifying specific cancer subtypes and guiding treatment in the age of personalized or precision medicine.

Molecular profiles with bimodal expression provide excellent candidates for biomarkers because the modes can be used to classify samples into two distinct expression states. During the biomarker discovery process, a bimodal expression profile may be considered meaningful when the modes of expression correspond with binary biological phenotypes, such as healthy and disease states. A biomarker that is deemed meaningful then needs follow up studies to determine the sensitivity and specificity before it could be considered accurate and reliable for practical application. However, it is typically rare that a molecule associated with a disease phenotype can be assayed with the sensitivity and specificity required for a clinical diagnostic test.^3^ One advantage of biomarker candidates with bimodal profiles at the transcript level is that they may be easily translated to the protein level and IHC staining, for a greater variety of available assays. Bimodal transcript expression typically corresponds with membrane and extracellular proteins, where molecules used as cancer biomarkers primarily localize.^3^,^4^ A variety of available assays may need to be evaluated at the gene or protein level before an adequate reliability is obtained. Estrogen receptor, for example, has served as an important biomarker in breast cancer, but assays have had varying success and some but not all assays capture a bimodal distribution.^5^,^6^

The method presented in Wang et al was applied to the MDA133 breast cancer microarray dataset previously published by Hess et al.^1^,^7^ The MDA133 microarray dataset is accompanied by clinical information including immunohistochemistry (IHC) scores for markers currently used to evaluate breast cancer, including estrogen receptor (ESR1), progesterone receptor (PGR), and human epidermal growth factor receptor 2 (HER2, or ERBB2). These markers define subcategories of breast cancer that differ in response to therapy as well as overall survival.^2^ IHC scores for these proteins are graded by pathologists and used to guide the diagnosis and treatment of breast cancer subtypes, and there is evidence that transcript profiles for these markers correlate well with protein measures.^8^,^9^ The IHC profiles of these markers, based on the dataset from Hess et al available at http://bioinformatics.mdanderson.org/pubdata.html, follow a bimodal distribution (Fig. 1).^7^ The bimodal distribution is suitable for defining a cut point between the two modal peaks. The cut-point used for the IHC scores corresponding to each molecule in the Hess et al^7^ dataset demonstrate this, and are identified with the dashed vertical red line in Figure 1. With the established bimodal distributions of IHC scores for these markers, Wang et al^1^ investigated the gene expression profiles and Bimodality Index for these three genes, and found that they all had high scores for bimodality. The bimodal expression profiles for these three transcripts, using log_2_ transformed data from Hess et al^7^ are shown in Figure 2. The software package for computing the bimodality index also provides parameters for the bimodal mixture distributions, which were used to define marker classification thresholds shown as dashed vertical red lines in Figure 2. While the authors only commented on the proportion of samples represented by each mode, the mode of expression from the transcript profile is shown by the degree of shading to correlate well with the mode of expression from the IHC score. This serves not only as a validation for the bimodality index in real data, but also demonstrates that an automated transcript-based assay may be an attractive alternative to manually scored IHC.

The correspondence between the protein and transcript level expression for these three markers shows much promise for the application of the bimodality index to biomarker discovery. However, a bimodal expression profile alone does not imply that a molecule will have a meaningful correlation with a biological or clinical variable of interest. The authors provide an example of a problematic candidate, where the marker creatine kinase, brain (CKB) has a strong bimodal profile that appears to be associated with breast cancer, and furthermore, advanced stage of disease, but provided limited value as a prognostic marker in this disease.^10^ Recognizing that many biomarker candidates will turn out to be false positives emphasizes the advantage to using a score such as the bimodality index, in that it relates directly to power and sample sizes and provides a ranking system for the systematic assessment and validation of biomarker candidates. This aspect should prove valuable in efficiently evaluating biomarker candidates from discovery through validation to establish clinically relevant molecules and assays.

## Figures and Tables

**Figure 1.**
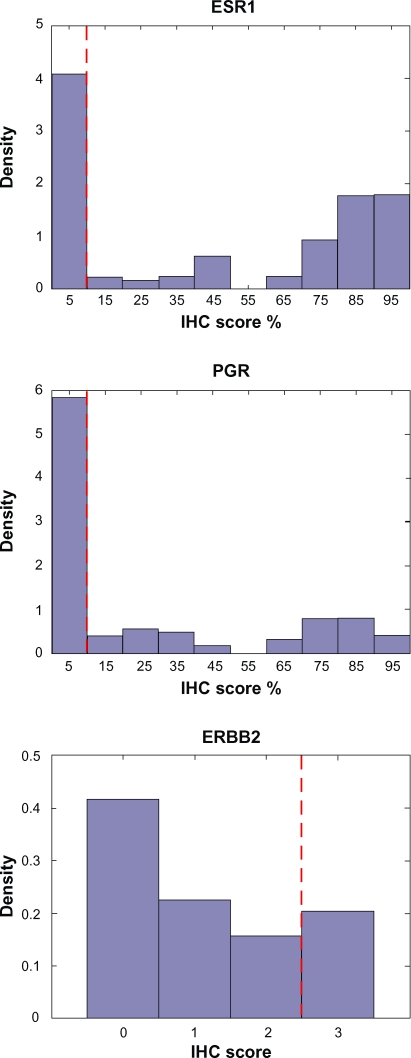
**Histograms representing IHC scores for ESR1, PGR, and ERBB2.** These three IHC markers appear as bimodal distributions in the MD Anderson 133 sample dataset. Dashed vertical red lines define thresholds for dichotomizing values as marker-positive and marker-negative.

**Figure 2.**
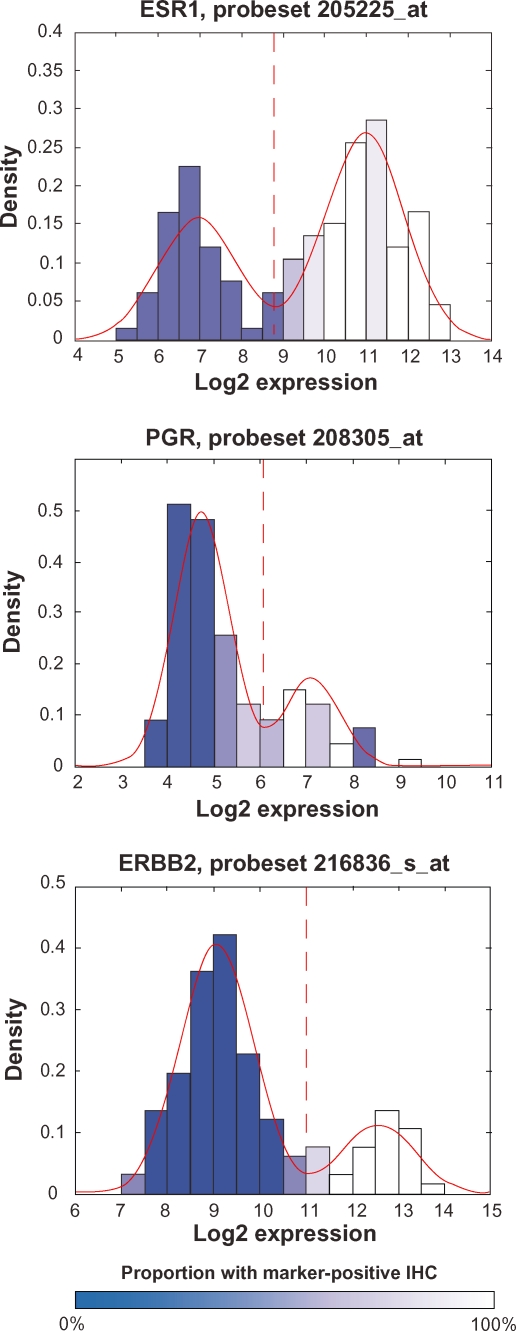
**Histograms representing transcript level distributions for the ESR1, PGR, and ERBB2 genes.** The transcripts for these three genes have bimodal distributions with the dashed vertical line representing the classification threshold between the two modes. The histogram shading represents the proportion of marker-positive IHC scores in each bin (Dark blue corresponds to marker-negative IHC and white corresponds to marker-positive IHC). The solid red line represents the bimodal distribution density estimate based on parameters from the bimodality index software package.
